# Spray-synthesized organic composite/hydroxyapatite coating on magnesium alloys with enhanced corrosion resistance

**DOI:** 10.3389/fchem.2025.1566676

**Published:** 2025-04-02

**Authors:** Guoqiang Wang, Yi Wei, Jinquan Hong, Jiangquan Lv

**Affiliations:** ^1^ Minjiang Collaborative Center for Theoretical Physics, College of Physics and Electronic Information Engineering, Minjiang University, Fuzhou, China; ^2^ College of Electronics and Information Science and Organic Optoelectronics Engineering Research Center of Fujian’s Universities, Fujian Jiangxia University, Fuzhou, China

**Keywords:** spray, organic composite/hydroxyapatite coating, corrosion resistance, magnesium, layer-by-layer assembly

## Abstract

The development and production of anti-corrosive coatings are critical for medical implants, particularly those that utilize composite coatings made from both flexible organic materials and rigid inorganic materials, which exhibit advantageous mechanical properties and resistance to corrosion. In this work, an organic composite/hydroxyapatite (HA) coating on magnesium alloys is fabricated through a two-step process, which involves the application of a spray technique for the organic silica composite [polyethyleneimine (PEI)/silica sol (Si)], followed by a hydrothermal treatment to deposit hydroxyapatite. The dense and tight layer-by-layer assembly of the PEI/Si/HA coating on the Mg substrate exhibited a corrosion current density of 10^−5.6^ A/cm^2^, significantly lower than that of HA and the Mg substrate. After immersion for 13 days, the PEI/Si/HA coatings demonstrated a minimal amount of H_2_ generation and negligible fluctuations in pH within the solution. Meanwhile, the hydrothermal PEI/Si/HA coatings exhibited significantly weaker corrosion resistance than the PEI/Si/HA coatings synthesized using the spray method. Both electrochemical dynamic data and structural characteristics demonstrate the enhanced corrosion resistance of organic composite/hydroxyapatite coatings, in which polymer chains provided more volume as a buffer for H_2_ molecules. This organic composite/hydroxyapatite coating on magnesium alloys exhibited huge potential applications in orthopedics.

## 1 Introduction

Magnesium (Mg) alloys have received widespread attention in the field of orthopedic implant materials due to their biodegradability, biocompatibility, and mechanical compatibility (Wang G. et al., 2024; Wang et al., 2022; Bryzgalov et al., 2024; Gao et al., 2024). However, rapid hydrogen evolution in the humoral environment accelerates the decline of the mechanical properties of Mg alloys, leading to unstable mechanical performance and affecting the effectiveness of internal fixation (Chen et al., 2024; Prithivirajan and Panigrahi, 2024; Osipenko et al., 2024; Hiromoto et al., 2021). Thus, surface modification is adopted to improve the corrosion resistance of Mg alloys.

Calcium phosphate (HA) coatings are considered appropriate structures due to their straightforward synthesis methods and effective corrosion resistance (Mei et al., 2024; Yeganeh and Mohammadi, 2018; Farshid et al., 2024). However, the mismatch in Young’s modulus at the interface between hydroxyapatite (HA) coatings (12–44 GPa) and magnesium metal (29 GPa) leads to stress concentration and coating fracture, ultimately resulting in diminished corrosion resistance (Yang et al., 2023a; Yang et al., 2023b; Sun et al., 2023; Akram et al., 2023; Fan et al., 2023). Therefore, it is necessary to modify HA coatings to address this mismatch. Zeng’s group combined polyelectrolytes [polyethyleneimine (PEI), polystyrene sulfonate (PSS), polyacrylic acid (PAA), and ammonium phosphate (PPA)] with HA coatings to enhance corrosion resistance, demonstrating the benefits of the interaction forces between the functional groups and Ca^2+^ ions (Kausar, 2024; Jiang et al., 2023). Chu et al. further synthesized multilayered PEI/PAA-HA coatings using a dip-coating method, in which the multilayered PEI/PAA played an important role in initiating the nucleation of HA and alleviating the lattice mismatch between the Mg alloys and the HA coating (Yang et al., 2023a). This indicates that organic/inorganic coatings have significant potential to address lattice mismatch and enhance the corrosion resistance of Mg alloys. Although progress has been made, there is still a need to explore more suitable methods and stable structures for HA coatings.

Spray application has garnered increasing attention for synthesized coatings due to its ease of operation and higher efficiency, resulting in coatings that are uniform and smooth (Cao et al., 2024; Niverty et al., 2024; Mengiste et al., 2024; Poblano-Salas et al., 2024; Srichen et al., 2024). This method uses external forces to either press or draw the coating from a container, creating a mist that adheres to the object’s surface. Gas spraying employs low pressure generated by compressed air expelled from a nozzle to extract the coating from the container. Once the ink is drawn out, it is atomized into a mist by the airflow and subsequently adheres to the object’s surface. The spray coating technique has been widely adopted in various fields, including aviation, military applications, and solar cell production, to create thin films that are firmly bonded to the substrate, enhance performance, and provide protection to the substrate (Meng et al., 2024; Yang et al., 2024a; Yao et al., 2024).

In the summary above, we introduced the spray methods to prepare an organic composite/hydroxyapatite coating on magnesium alloys (PEI/Si/HA). The cationic polymer polyethyleneimine and the anionic silica sol (Si) were sprayed onto the magnesium alloys. After repeating the spraying process three times, hydroxyapatite was deposited through a hydrothermal method. The resulting PEI/Si/HA coating on the magnesium substrate is dense and tightly assembled layer-by-layer. It exhibited a corrosion current density of 10^−5.6^ A/cm^2^, which is lower than that of HA and the magnesium substrate. Additionally, there was a minimal generation of hydrogen gas and negligible fluctuations in pH levels in the solution after immersion for 13 days. Both the electrochemical dynamic data and structural characteristics demonstrate the enhanced corrosion resistance of the organic composite/hydroxyapatite coatings, wherein the polymer chains provide additional volume to buffer H_2_ molecules. The organic composite/hydroxyapatite coating applied to magnesium alloys demonstrates significant potential for applications in the field of orthopedics.

## 2 Experimental section

### 2.1 Materials and reagents

AZ31B magnesium alloy sheets were purchased from Dongguan Qihao Metal Materials Co., Ltd., China, consisting of Al: 2.9 wt%, Zn: 0.93 wt%, Mn: 0.36 wt%, Mg remaining components. Tetraethyl orthosilicate, PEI (MW 10000 Da), Ca(NO_3_)_2_⋅4H_2_O, NH_4_OH, and NaH_2_PO_4_ were purchased from Shanghai Aladdin Biochemical Technology Co., Ltd. All reagents were purchased without pretreatment. DI water was used for all experiments.

### 2.2 Synthesis materials

#### 2.2.1 Synthesis of HA coatings:

The Mg alloy was polished using 300-grit and 2500-grit sandpaper to remove the oxide layers. Subsequently, it was soaked in a NaOH solution for 30 min. A mixture of 8.4 mM/L NaH_2_PO_4_ 2H_2_O (20 mL) and 14 mM/L Ca(NO_3_)_2_ 4H_2_O (20 mL) was prepared and stirred. Then, 20 mL of a 4 mM/L sodium bicarbonate (NaHCO_3_) solution was added to the mixture. The resulting solution and the pretreated magnesium plate were placed in a hydrothermal kettle and maintained at 150°C for 4 h. Finally, the HA coating on Mg plates was obtained.

#### 2.2.2 Synthesis of PEI/Si/HA coatings

A 1 mL aliquot of NH_4_OH was added to 3 mL of tetraethyl orthosilicate while stirring for 1 h to obtain a silicon sol. Subsequently, 0.2 mL of a polyethyleneimine aqueous solution (1 g/L) was sprayed onto the pretreatment magnesium alloys. After drying, 0.2 mL of the silicon sol was sprayed onto the PEI coatings. This spraying process was repeated three times (PEI/Si). Following this, the sprayed alloy was placed in a hydrothermal kettle with a mixture of the Ca-P solution (see above “synthesis of HA coatings”), and the PEI/Si/HA coatings on the magnesium substrate were obtained. The PEI/Si/HA-1 coatings were produced by cycling the process once, while the other steps remained the same as described above. The PEI/Si/HA-5 coatings were obtained by cycling the process five times.

#### 2.2.3 Synthesis of PEI/Si/HA coatings:

A solution of 4 mM/L NaH_2_PO_4_ 2H_2_O (20 mL) and 14 mM/L Ca(NO_3_)_2_ 4H_2_O (20 mL) was mixed and stirred. Then, 20 mL NaHCO_3_ (4 mM/L) solution was added to the above solution. Silicon sol and PEI solution were added to the above solution in sequence. After stirring, the mixture solution was moved to a hydrothermal kettle and kept at 150°C for 4 h.

### 2.3 Structural characteristics

The structures of the obtained materials were characterized using X-ray diffraction (XRD, D/max 2550 V) with Cu Kα radiation (λ = 0.154178 nm) over a range of 10°–70°. Scanning electron microscopy (SEM, S-4800, Hitachi, Japan) was employed to analyze the morphology. Fourier transform infrared (FTIR) spectroscopy in the range of 400–4,000 cm^−1^ was utilized to observe the functional groups.

### 2.4 Electrochemical measurements

A CHI660e electrochemical working station (CH Instruments) was used with a typical three-electrode s stem, in which a Pt mesh, an Ag/AgCl (3 M KCl) electrode, and a 0.5 cm^2^ Mg substrate were used as the counter electrode, reference electrode, and work electrode, respectively. Experiments were performed in Hank’s solution using linear sweep voltammetry (LSV) at a scan rate of 5 mV s^−1^. The composition of the solution is shown in Supplementary Table S1 (Abidin et al., 2011; Ghoneim et al., 2010). Electrochemical impedance spectroscopy (EIS) was performed to obtain the resistance of the reaction.

### 2.5 Evaluation of degradation

Each sample was submerged in Hanks’ solution at 37°C for 7 days to assess their corrosion performance by measuring the cumulative hydrogen evolution. After this period, the specimens were removed and promptly rinsed with water. The observable surfaces were then assessed through both macroscopic examination and scanning electron microscopy.

### 2.6 Tensile test

A tensile test was conducted using a universal tensile testing machine. The material was prepared in dimensions of 5 cm × 0.5 cm (±0.1 cm) and secured in the fixtures. The fixtures moved at a rate of 1 mm/min.

## 3 Results and discussion

**FIGURE 1:**
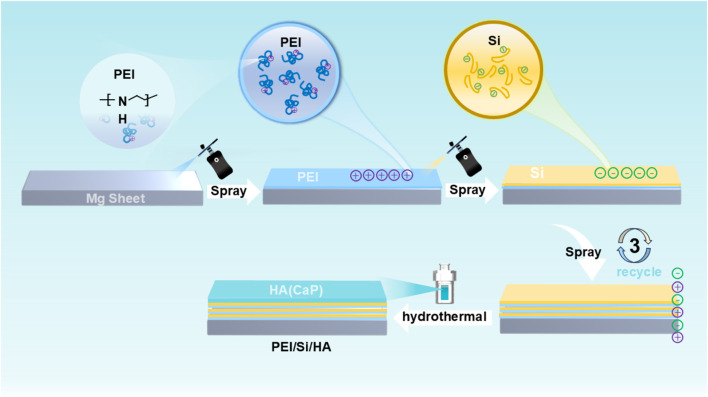
Schematic method of PEI/Si/HA.

**FIGURE 2:**
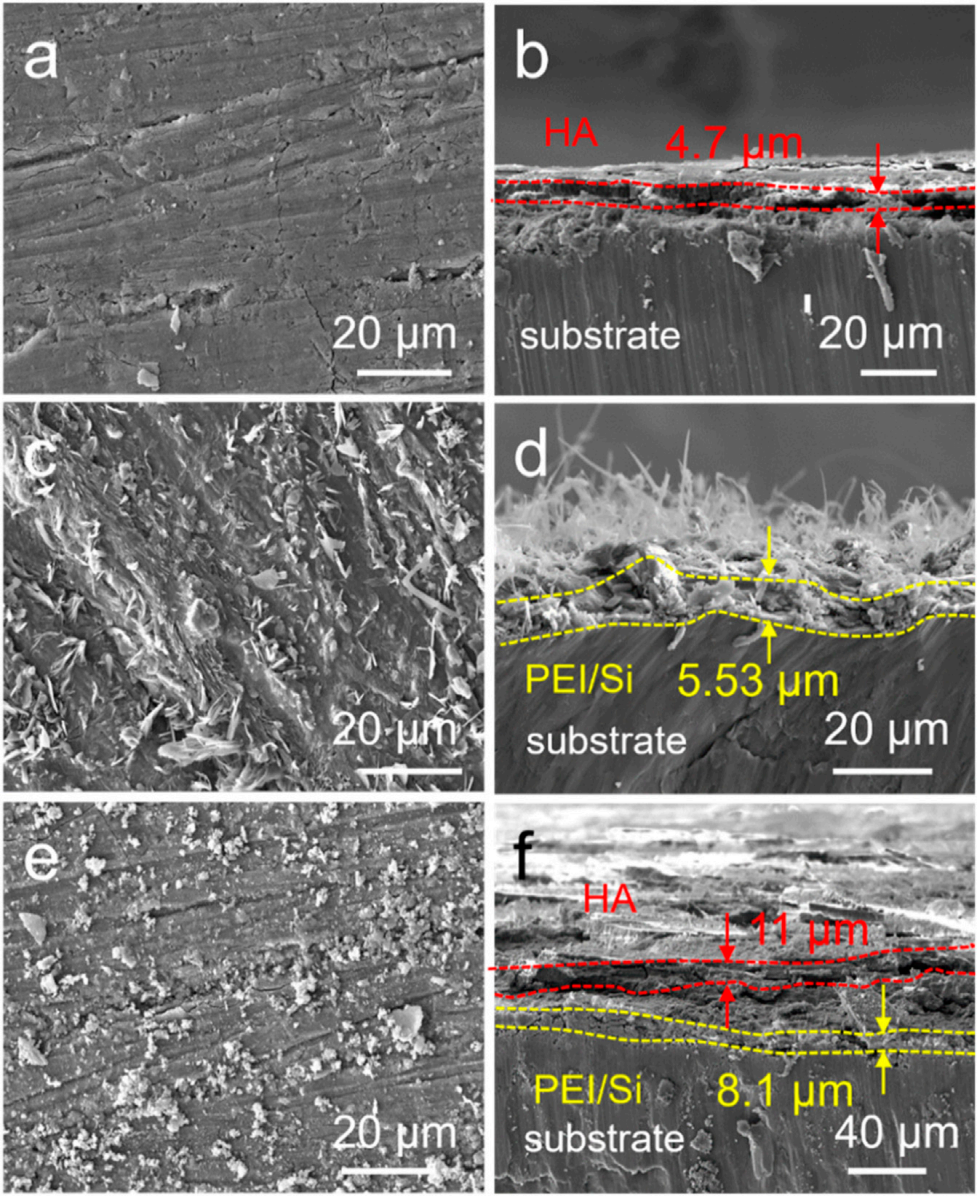
SEM images of **(a)** HA, **(c)** PEI/Si, and **(e)** PEI/Si/HA. Cross-sectional images of **(b)** HA, **(d)** PEI/Si, and **(f)** PEI/Si/HA.

**FIGURE 3:**
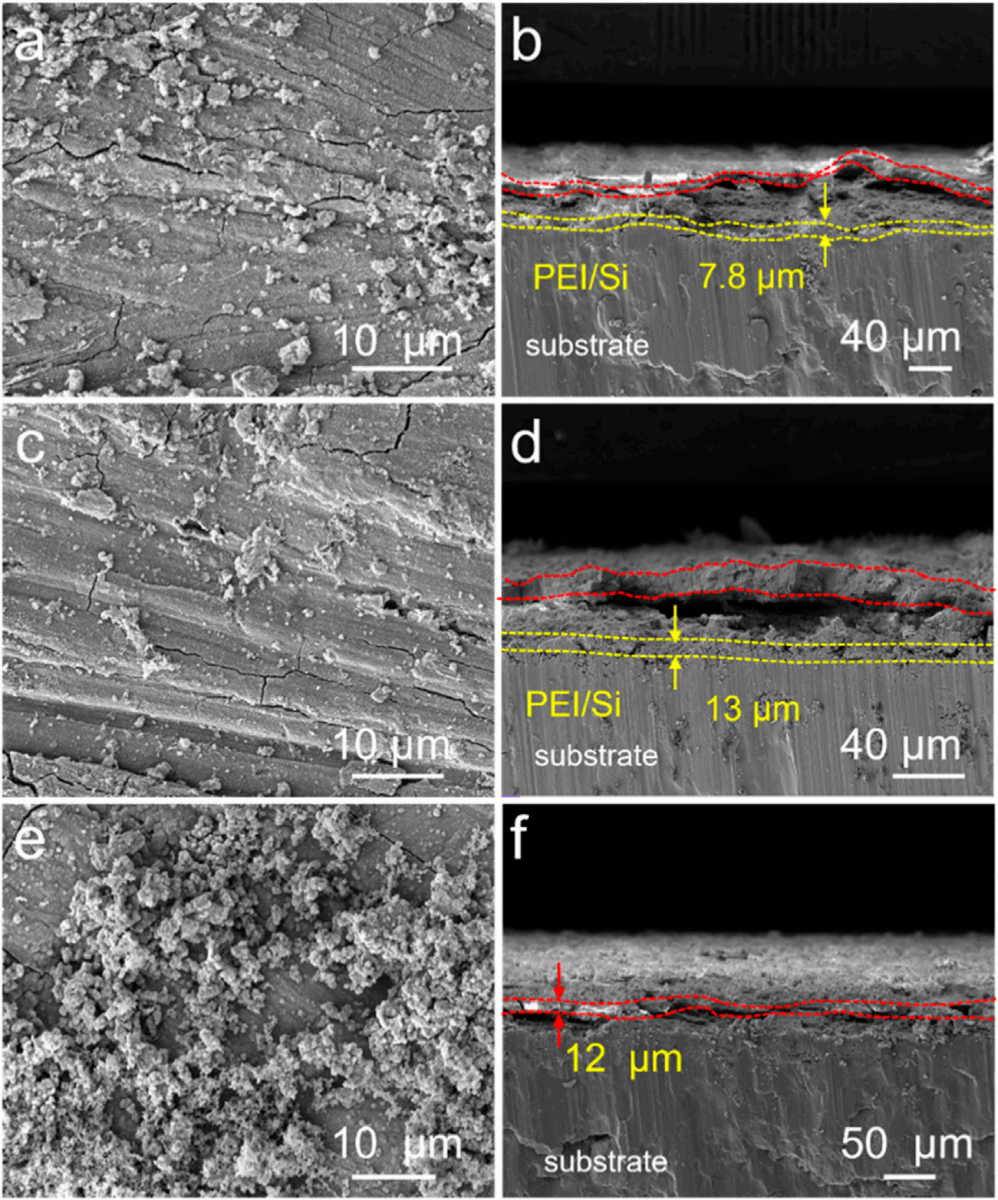
SEM images of **(a)** PEI/Si/HA-1, **(c)** PEI/Si/HA-5, and **(e)** PEI/Si/HA/Mg. Cross-sectional images of **(b)** PEI/Si/HA-1, **(d)** PEI/Si/HA-5, and **(f)** PEI/Si/HA/Mg.

The PEI/Si/HA multilayered coating was prepared using layer-by-layer (LbL) assembly on the Mg alloy, as illustrated in Figure 1. As shown, the cationic polymer PEI was initially applied to magnesium alloys via a spraying method. The anionic silica sol (TEOS) was further sprayed on PEI coating. This spraying procedure was repeated three times, after which hydroxyapatite was synthesized on the surface using a hydrothermal method. The PEI/Si/HA coating was formed on the Mg substrate. A scanning electron microscope was utilized to observe the surface morphology and thickness of the formed film. Figure 2A shows a dense HA film of HA coatings on the Mg substrate. The PEI/Si sprayed on Mg alloys was synthesized and exhibited dense film with arborization morphology on the surface (Figure 2C). The PEI/Si/HA coatings are shown in Figure 2E in a dense film with an arborized morphology on the surface. The thickness of the films was explored by cross-sectional images (Figures 2B, D, F). The tight films demonstrated the successful layer-by-layer assembly coating on the Mg substrate, demonstrating an effective way to synthesize films by spray coating. Note that cracks on the PEI/Si/HA resulted from shearing during SEM sample preparation to expose the cross-section. In addition to the above, we also studied the morphology of the material obtained by spray coating different numbers of times. As shown in Figures 3A, B, the films with one spray coat (PEI/Si/HA-1) exhibited a thinner coating than the films that were sprayed three times (PEI/Si/HA) and five times (PEI/Si/HA-5 in Figures 3C, D). As shown, the number of sprayed cycles had no negative impact on the tightness between the HA and the substrate, as well as between HA and upper PEI/Si layers. The one-pot hydrothermal PEI/Si/HA/Mg (Figures 3E, F) displayed coatings that consisted of nanoparticles. This may result in many gaps, making it easier for electrolytes to enter the Mg surface through the gaps. The above data prove the multilayered PEI/Si/HA coating and the feasibility of synthetic pathways with spray.

**FIGURE 4:**
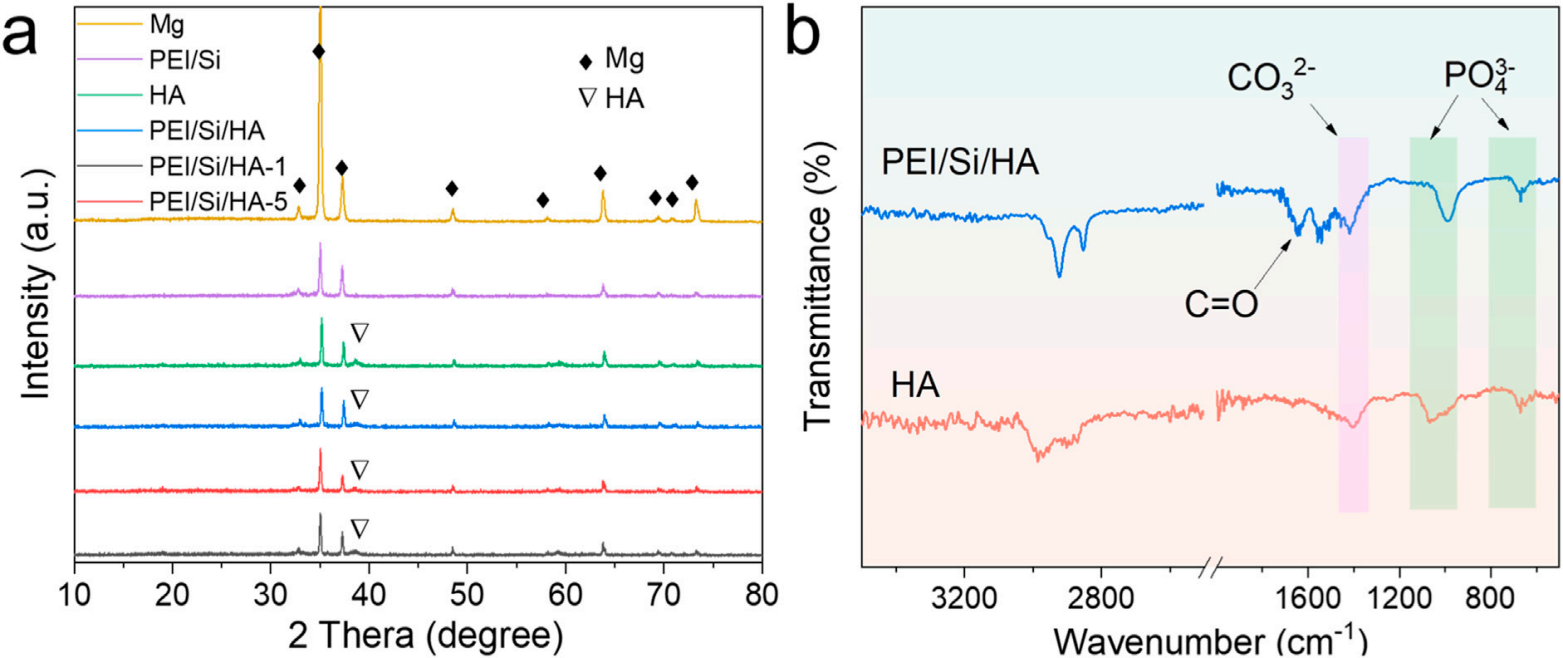
Structure of prepared films. **(a)** XRD patterns. **(b)** FTIR spectra.

X-ray diffraction (XRD) and Fourier transform infrared spectroscopy were conducted to explore the structural characteristics. As shown in Figure 4A, the peak of HA observed at 38.6° stemmed from hydroxyapatite, demonstrating the successful synthesis of HA coatings on the Mg alloy (Shen et al., 2017; Cui et al., 2020; Ju et al., 2023). No additional peaks appeared in the PEI/Si/HA, indicating the amorphous nature of the formed polymer films. The structure was further detected by FTIR, as shown in Figure 4B. The peaks at 603 cm^−1^ and 1,041 cm^−1^ are the typical bands of HA. The characteristic peak at 1,400 cm^−1^ is attributed to the CO_3_^2−^. HA easily adsorbed CO_2_ from the environment during preparation or storage. Part of the PO_4_^3−^ or OH^−^ in HA may be replaced by CO_3_^2−^, forming carbonate apatite (Ca_10_(PO_4_)_6-x_ (CO_3_^2−^)_x_ (OH)_2−x_). In PEI/Si/HA, the peak around 1700 cm^−1^ is attributed to the C=O bond, mainly belonging to PEI/Si layer (Khan and Awais, 2020; Mahmoodi et al., 2013; Xu et al., 2024; Yang et al., 2024b; Zeng et al., 2016). The above results demonstrated the formation of PEI/Si/HA coatings on Mg substrate.

**FIGURE 5:**
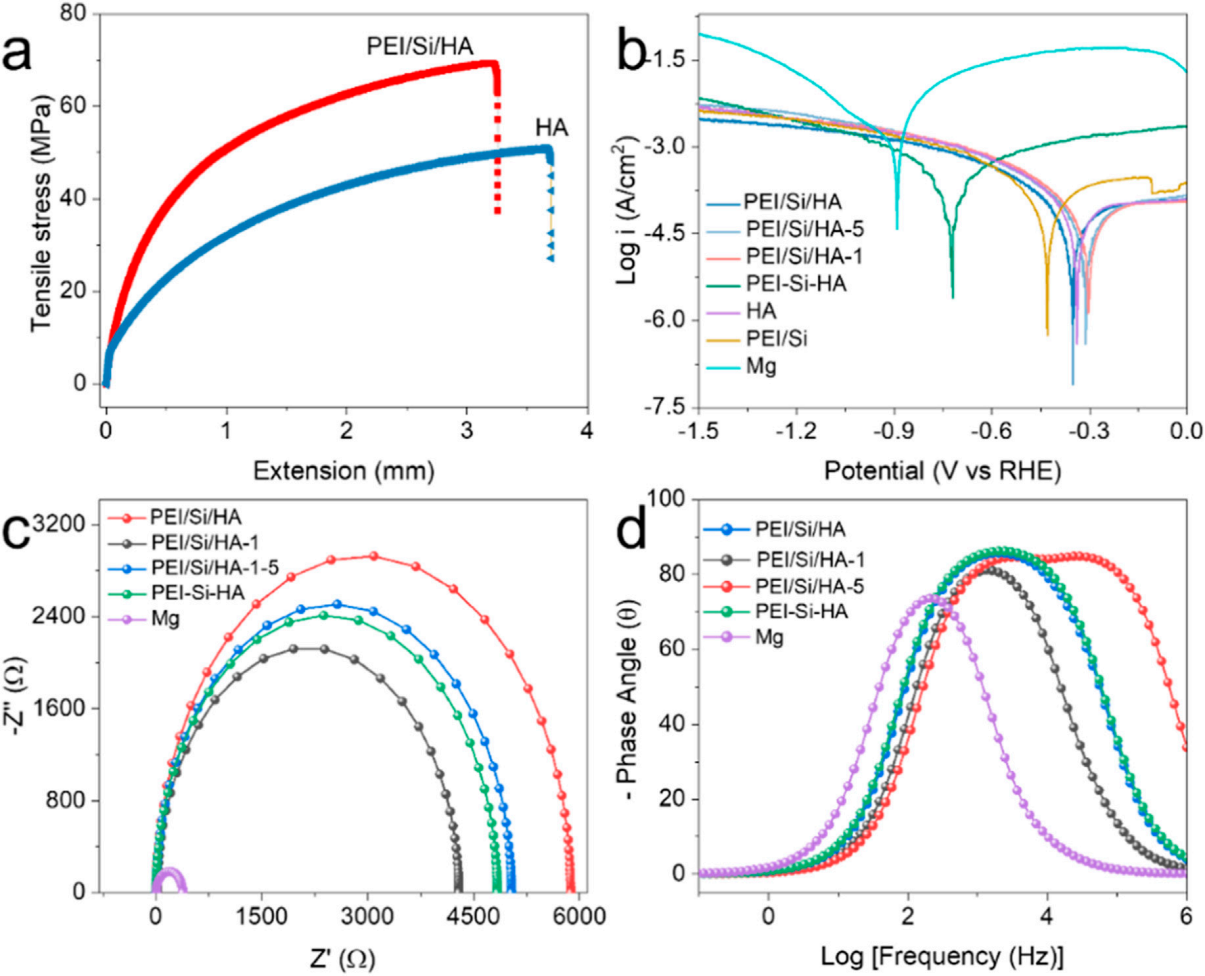
**(a)** Tensile–extension plots. **(b)** Polarization curves. **(c)** Nyquist plots. **(d)** Bode plots.

Figure 5A shows the interface bonding strength to observe the enhancement effect of coating modification on the substrate. The PEI/Si/HA exhibited a tensile stress of 69.3 Mpa, higher than HA (50.84 Mpa). In addition, Young’s modulus of PEI/Si/HA was higher than HA, indicating the greater stiffness of PEI/Si/HA. The improvement was related to the interaction forces from the specific functional groups (PO_4_^3−^ and OH^−^ in HA, -NH_2_ and -NH- in PEI, and -OC_2_H_5_ and Si-O in Si sol) and tight binding between HA and the Mg substrate and between PEI/Si and HA. The modified coating has great potential applications in orthopedics.

Corrosion resistance is an important parameter for evaluating the effectiveness of coating modification on magnesium substrates in simulated human bone environments. To demonstrate the corrosion rate of the material, the electrochemical polarization curves were tested. According to published literature (Shamsi et al., 2024; Yang et al., 2024; Shishir et al., 2024; Yang et al., 2024c), the lower the corrosion current and higher corrosion potential demonstrated the better the anti-corrosion effect. The I_corr_ was obtained from the cathodic polarization zone. As shown in Figure 5B and Supplementary Table S2, the corrosion current density (I_corr_) of PEI/Si/HA (10^−5.6^ A/cm^2^) was much lower than that of the Mg substrate (10^−3.2^ A/cm^2^), suggesting that the corrosion resistance of the substrate has been effectively increased by the optimized dense coating. Meanwhile, as the number of spray coatings increased, the current initially declined and subsequently rose, indicating that an appropriate thickness of the films was crucial for effective application. The PEI/Si/HA coatings displayed a corrosion current density of 10^−4^ A/cm^2^ at a corrosion potential (E_corr_) of −0.721 V vs. RHE, indicating a strong corrosion activity compared to the PEI/Si/HA coatings. It further demonstrated the benefits associated with the spray application technique.

**FIGURE 6:**
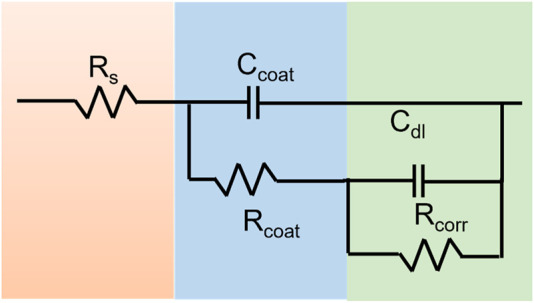
Equivalent circuit to fit impedance.

EIS measurements were used to characterize the resistance of materials. Nyquist plots and Bode plots are shown in Figures 5C, D, 6, and Supplementary Table S3, in which the PEI/Si/HA exhibited much higher impedance than that of HA and the Mg substrate. It confirmed that the coating produced through the spraying method significantly enhances the corrosion resistance of magnesium alloys. The impedance changed with the number of spray coatings, indicating that the coating thickness affects performance. Furthermore, the R_corr_ and R_coat_ values of the sprayed PEI/Si/HA coatings exhibited greater impedance than the PEI/Si/HA coatings synthesized through the hydrothermal process, which demonstrated superior corrosion resistance when synthesized using the spray method. The above experiments demonstrated that a dense PEI/Si/HA coating on an Mg substrate effectively decreases the corrosion rate, displaying great potential for application in orthopedics.

**FIGURE 7:**
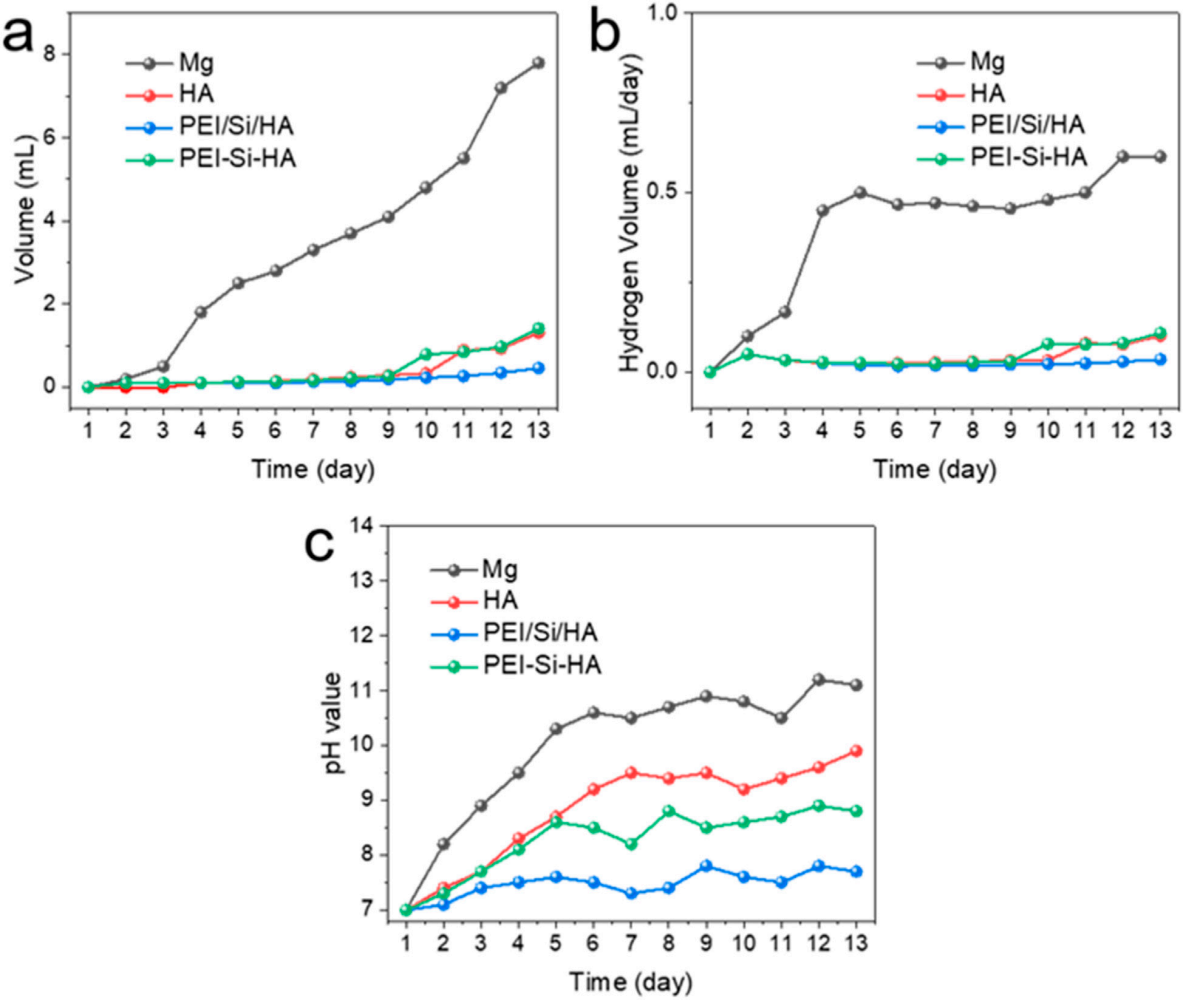
**(a)** Volume of H_2_. **(b)** H_2_ generation rate curves. **(c)** pH of the solution.

The stability of the substrate in solution is an important parameter for evaluating whether it can be applied in orthopedics. To test the long-term corrosion characteristics, prepared coatings were immersed in Hanks’ solution at room temperature (Li et al., 2022; Sun et al., 2019). The H_2_ production is shown in Figures 7A, B, displaying 0.46 mL of PEI/Si/HA coatings after immersion 13 days, lower than that of HA coatings (1.31 mL), PEI/Si/HA coatings (1.41 mL) (hydrothermal synthesized), and the Mg substrate (7.8 mL), respectively. The rate of H_2_ generation was 0.035 mL/day, which was lower than HA coatings (0.1 mL), PEI/Si/HA coatings (0.1 mL), and the Mg substrate (0.6 mL), respectively. It displayed strong inhibition of the solution and enhanced the anti-corrosion effect. The pH value in Figure 7C showed negligible fluctuation of PEI/Si/HA coatings, suggesting a slow hydrogen evolution reaction. The above results indicate that tightly combined PEI/Si and HA coatings could provide better barriers and effectively enhance the corrosion resistance of the substrate.

**FIGURE 8:**
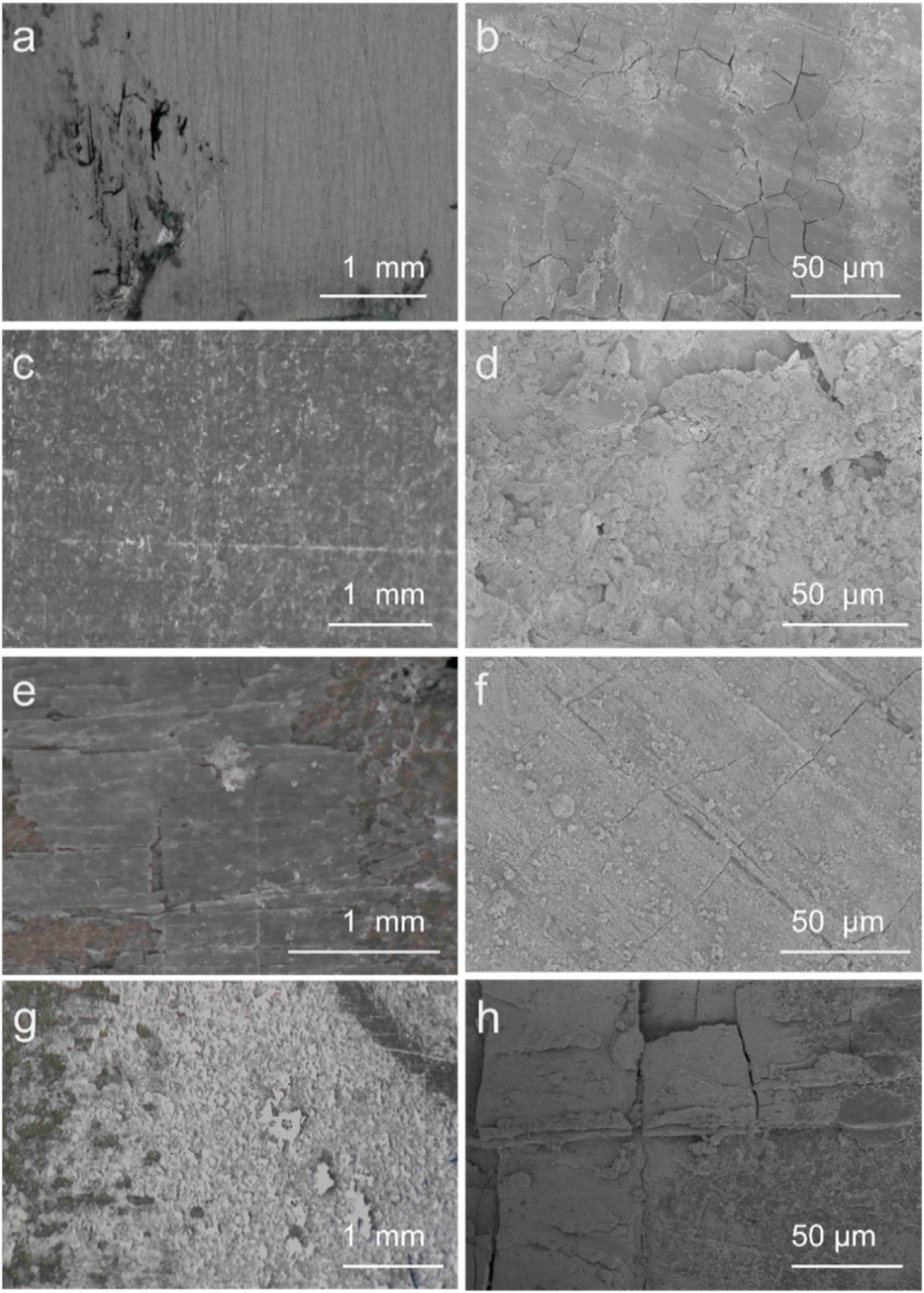
SEM images of **(a)** and **(b)** Mg, **(c)** and **(d)** PEI/Si/HA coatings, **(e)** and **(f)** HA coatings, **(g)** and **(h)** PEI/Si/HA coatings after immersion for 13 days.

After long-term immersion, the SEM was used to further survey the stability of coatings (Figure 8). After the immersion, the surface of Mg alloy exhibited severe corrosion and cracks, and HA coatings displayed peeling-off on the surface and cracks. The PEI/Si/HA had fewer cracks, indicating weak corrosion due to the solution. The SEM of PEI/Si/HA coatings (Figures 8G, H) displayed severe peeling and shedding. This may be due to the severe aggregation of nanoparticles in the Mg surface, resulting in a weak combination between the coatings and the Mg substrate. It further demonstrated the advantage of PEI/Si/HA coatings synthesized by the spray method. The tight combination of HA coatings and PEI/Si built a buffer layer, effectively reducing the probability of solution entry and corrosion.

**FIGURE 9:**
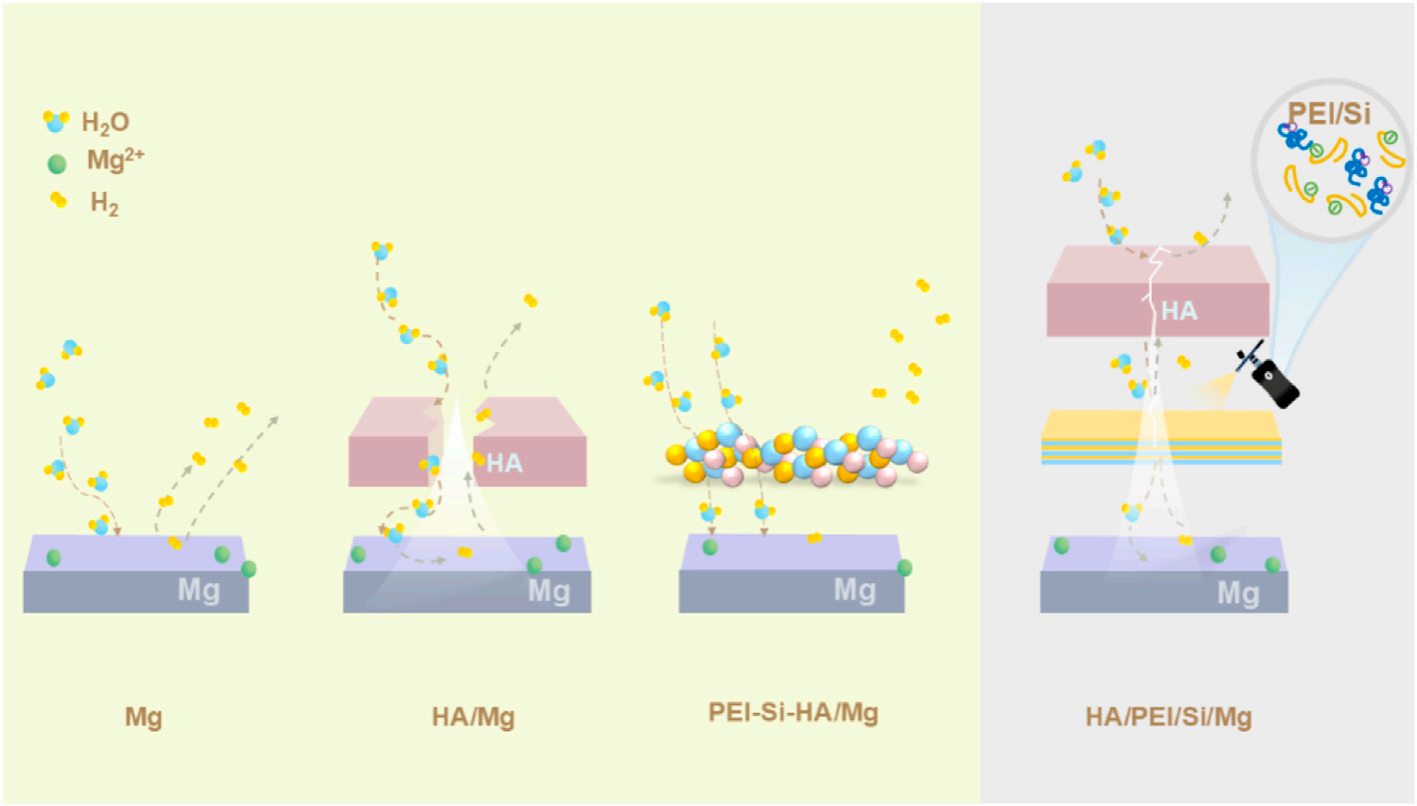
Corrosion mechanism of PEI/Si/HA coatings. **(a)** Mg. **(b)** HA/Mg. **(c)** PEI/SI/HA/Mg. **(d)** HA/PEI/Si/Mg.

The anti-corrosion mechanism of PEI/Si/HA coatings on Mg alloy is explained in Figure 9. During the orthopedic implant application of Mg substrate (Figure 9A), water molecules in the solution of the human body diffuse inward into the surface of Mg alloy, resulting in Mg corrosion and H_2_ emission (Wang S. et al., 2024; Zhao et al., 2018; Hou et al., 2024). The HA coatings in Mg alloys, because of the mismatch between HA and Mg, still suffer cracking, causing cracks in the HA layer (Figure 9B). The severe aggregation of nanoparticles in hydrothermal PEI/Si/HA coatings (Figure 9C) resulted in a weak combination between coatings and Mg substrate, making it easier for electrolytes to enter the Mg surface. Introducing organic PEI/Si coatings between HA and Mg relieved the problem of lattice pressure and strengthened the structure stability (Figure 9D). The HA coatings on the surface played the role of the first barrier, and the organic PEI/Si multilayer supplied a dual protection layer. In addition, the polymer chains provided more volume as a buffer for H_2_ molecules. Therefore, the organic PEI/Si and hydroxyapatite coating collaborated to enhance the corrosion resistance of Mg alloys.

## 4 Conclusion

An organic composite/hydroxyapatite coating on Mg alloys is prepared by spraying for organic composite (PEI/Si) and hydrothermal for HA. SEM images show the dense and tight layer-by-layer assembly of PEI/Si/HA coating (spray coated) on an Mg substrate, demonstrating the effectiveness of the spray method in preparing coating. The corrosion current density of Mg alloys was decreased to 10^−5.6^ A/cm^2^ after PEI/Si/HA coating. PEI/Si/HA coatings exhibited a small amount of H_2_ generation and negligible pH fluctuation in solution after immersion in solutions. The hydrothermal PEI/Si/HA coatings demonstrated markedly inferior corrosion resistance compared to the PEI/Si/HA coatings produced through the spray technique. The findings offer valuable perspectives on the design and production of organic/inorganic coatings intended for biomedical applications, as well as directions for future investigations related to the clinical utilization of magnesium alloys in orthopedics and other medical disciplines.

## Data Availability

The original contributions presented in the study are included in the article/Supplementary Material; further inquiries can be directed to the corresponding authors.
