# Supplementary material for: Spray-synthesized organic composite/hydroxyapatite coating on magnesium alloys with enhanced corrosion resistance
Source: Front Chem. 2025 Apr 2;13:1566676. doi: 10.3389/fchem.2025.1566676 (PMC12000018; doi:10.3389/fchem.2025.1566676)
Supplement: Supplementary file 1 [file Table1.docx]

Supplementary Material

**Spray-Synthesized Organic Composite/Hydroxyapatite Coating on Magnesium Alloys with Enhanced Corrosion Resistance**

**Guoqiang Wang ^1,2, +^, Yi Wei ^2, +^, Jinquan Hong ^1,^ * and Jiangquan Lv ^2,^ ***

^1^ Minjiang Collaborative Center for Theoretical Physics, College of Physics and Electronic Information Engineering, Minjiang University, Fuzhou, China

^2^ College of Electronics and Information Science & Organic Optoelectronics Engineering Research Center of Fujian's Universities, Fujian Jiangxia University, Fuzhou, China

^+^equal contribution

*** Correspondence:**

Corresponding Author: Jiangquan Lv, Jinquan Hong

e-mail: [jqlv@fjjxu.edu.cn](mailto:jqlv@fjjxu.edu.cn), [jqhong@mju.edu.cn](mailto:jqhong@mju.edu.cn)

Table S1 Concentration of reagents in Hank’s solution.

| Reagents | Concentration (g/L) |
| --- | --- |
| NaCl | 8 |
| KCl | 0.4 |
| NaHCO_3_ | 0.35 |
| MgSO_4_ 7H_2_O | 0.2 |
| MgCl_2_ 6H_2_O | 0.1 |
| CaCl_2_ | 0.14 |
| Na_2_HPO_4_ 2H_2_O | 0.06 |
| KH_2_PO_4_ | 0.06 |
| Glucose | 1 |

Table S2 corrosion current density (J_corr_) and corrosion potential (E_corr_).

| Sample | Ecorr (V vs RHE） | Icorr (A/cm^2^) |
| --- | --- | --- |
| Mg | -0.891 | 10^-3.2^ |
| HA/Mg | -0.338 | 10^-5.1^ |
| PEI/Si/HA/Mg | -0.35 | 10^-5.6^ |
| PEI/Si/HA/Mg-1 | -0.302 | 10^-4.9^ |
| PEI/Si/HA/Mg-5 | -0.313 | 10^-5.0^ |
| PEI/Si/Mg | -0.43 | 10^-4.4^ |
| Hydrothermal PEI-Si-HA | -0.721 | 10^-4.0^ |

Table S3 Resistance of coating (R_coat_) and corrosion (R_corr_).

| Sample | R_coat_ (Ω) | R_corr_ (Ω) |
| --- | --- | --- |
| Mg | 367 | 4.619 |
| PEI/Si/HA/Mg | 5462 | 221 |
| PEI/Si/HA/Mg-1 | 4252 | 62.2 |
| PEI/Si/HA/Mg-5 | 4485 | 68.31 |
| Hydrothermal PEI-Si-HA | 4752 | 72.14 |
